# Determinants of Compliance to Enhanced Recovery Protocol After Emergency Laparotomy

**DOI:** 10.1002/wjs.70312

**Published:** 2026-03-25

**Authors:** Marco Ceresoli, Chiara Fumagalli, Alan Biloslavo, Antonio La Greca, Michele Carlucci, Giovanni Pesenti, Savino Occhionorelli, Pietro Bisagni, Carlo Feo, Dario Tartaglia, Dario Parini, Matteo Runfola, Riccardo Somigli, Diego Visconti, Diego Mariani, Diletta Cassini, Andrea Mingoli, Marco Braga, the Study Collaborative Group

**Affiliations:** ^1^ University of Milano‐Bicocca School of Medicine and Surgery Monza Italy; ^2^ General and Emergency Surgery Fondazione IRCCS San Gerardo dei Tintori Monza Italy; ^3^ Department of General Surgery Cattinara University Hospital ASUGI Trieste Italy; ^4^ Dip.to Scienze Mediche e Chirurgiche UOC Chirurgia d'Urgenza e Trauma Fondazione Policlinico Universitario A. Gemelli IRCCS Roma ‐ Università Cattolica del Sacro Cuore Milano Italy; ^5^ Department of Emergency and General Surgery IRCCS San Raffaele Hospital Milano Italy; ^6^ UOC Chirurgia Generale e d'Urgenza Dip. chirurgico H. A. Manzoni Lecco Italy; ^7^ s.s.d. Emergency surgery Surgical Department University of Ferrara Ferrara Italy; ^8^ Department of Surgery ASST Lodi Lodi Italy; ^9^ UOC Chirurgia Generale Provinciale Azienda USL di Ferrara ‐ Università di Ferrara Ferrara Italy; ^10^ General Emergency Surgery Unit and Trauma Center Pisa University Hospital Pisa Italy; ^11^ General Surgery Department Santa Maria della Misericordia Hospital Rovigo ‐ ULSS 5 Rovigo Italy; ^12^ General and Emergency Surgery Unit Department of Emergency ARNAS “G. Brotzu” Cagliari Italy; ^13^ Dipartimento Chirurgia usl toscana centro‐ ospedale Santa Maria Nuova Firenze Italy; ^14^ Chirurgia Generale d’Urgenza e PS AOU Città della Salute e della Scienza Torino Italy; ^15^ General Surgery Ospedale di Legnano ASST ovest Milanese Legnano Italy; ^16^ General Surgery ASST Milano‐Nord Sesto San Giovanni Italy; ^17^ President of the Italian Society of Emergency Surgery and Trauma (SICUT) Roma Italy

**Keywords:** emergency general surgery, enhanced recovery protocol, ERAS, perioperative care

## Abstract

**Background:**

Enhanced recovery protocols (ERP) are comprehensive, evidence‐based approaches aimed at accelerating patient recovery and improving surgical results; increasing evidence exists about their adoption in emergency laparotomy. The study aimed to evaluate the compliance to the proposed postoperative pathway and the determinants of late recovery.

**Methods:**

This is a multicenter observational prospective study involving 13 Italian centers. Inclusion criteria targeted adults undergoing emergency surgery for intestinal occlusion or perforation. The primary end point was the early recovery rate (discontinuation of intravenous fluids and the initiation of oral intake on postoperative day three) and its determinants. The secondary end point were reasons for uncompliance to postoperative ERP items.

**Results:**

Between March 2023 and March 2024, 760 patients were recruited and analyzed, 60.2 with intestinal obstruction and 39.8 with intra‐abdominal infections. Recovery was achieved by 53.7% of patients on postoperative Day 3. Among determinants, clinical frailty and ASA status negatively correlated with recovery whereas the absence of surgical drains, anesthetic depth monitoring and intraoperative goal‐directed fluid therapy positively correlated with recovery. An analysis of the reasons for noncompliance with postoperative items revealed that aside from postoperative complications, the most frequently cited reason was protocol deviation for clinical decision, accounting for approximately 10%–15% of noncompliance for each item.

**Conclusions:**

This study showed that half of the patients reached the recovery goal on postoperative Day 3 and that early recovery after surgery is influenced both by intrinsic patient factors and by adherence to ERP strategies. Future research should prioritize strategies to improve ERP adherence and postoperative compliance.

## Graphical abstract

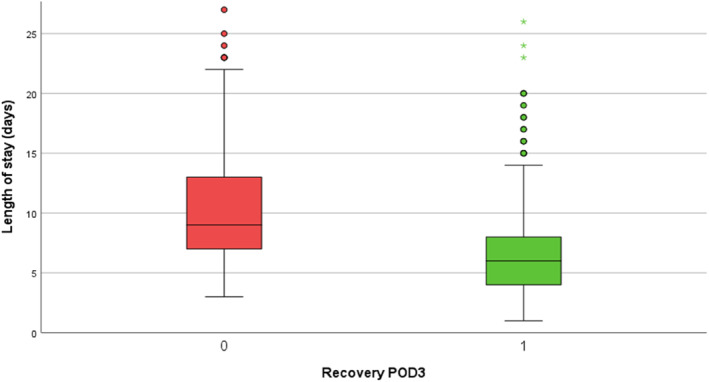

## 1 Background

Enhanced recovery protocols (ERP) are comprehensive and evidence‐based approaches. In recent years, these protocols have revolutionized perioperative care in elective surgery by promoting early mobilization, reduced perioperative stress, and faster return of gastrointestinal function [1]. These pathways have consistently demonstrated reductions in length of hospital stay, complication rates, and healthcare costs across various elective procedures [2]. They have been widely adopted as standard care in elective surgeries, included among elderly and high‐risk populations. However, the implementation of ERP principles in the emergency setting remains limited and under‐investigated, despite growing interest and promising data [3].

One of the core challenges in the emergency setting is the heterogeneity of patient conditions, combined with significant comorbidities and physiological derangements such as sepsis, hypovolemia, and metabolic abnormalities on admission. These factors may hinder the application of standardized recovery protocols and contribute to delayed postoperative recovery, making the consistent application of ERPs more complex and challenging [4]. Nonetheless, recent studies suggest that selected ERP components—such as avoidance of nasogastric tubes, early enteral nutrition, and multimodal analgesia—can be effectively adopted in emergency surgery with favorable outcomes, as also recommended by the ERAS society guidelines [5, 6, 7]. Despite these advances, there remains substantial variability in protocol designs across studies, and adherence to individual ERP components is often underreported, with most research focusing instead on postoperative results.

In an earlier study, our group demonstrated the feasibility of applying ERP principles in emergency cases, although many elements were not widely implemented. Following this initial experience, we launched a structured educational initiative across participating hospitals to promote consistent application. This led to further refinement of the protocol prior to initiating the present investigation [8]. There is currently limited evidence identifying which specific patient‐ or process‐related factors are most predictive of early postoperative recovery in the emergency setting. Understanding these factors is essential to tailor enhanced recovery protocols and to guide clinical decision‐making.

The primary objective of this study was to evaluate the rate of recovery on the third postoperative day and its determinants in patients undergoing emergency surgery for bowel perforation or obstruction. The secondary objective were the reasons for noncompliance to the proposed postoperative enhanced recovery pathway.

## 2 Material and Methods

This is a secondary analysis of a multicenter, prospective, and observational study conducted across 13 high‐volume Italian hospitals, all with established experience in applying enhanced recovery pathways (ERPs). The protocol focuses on optimizing patient care before, during, and after surgery was described elsewhere [9]. Eligible patients were adults undergoing emergency surgery for gastrointestinal obstruction or perforation. Exclusion criteria included the presence of septic shock at admission, emergency surgery due to complications following elective procedures, operative endoscopy, or diagnostic interventions. Patients who declined to participate were also excluded.

Additionally, patients requiring mechanical ventilation and/or vasopressor support for more than 24 h postoperatively, as well as those undergoing damage control surgery with an open abdomen, were withdrawn and excluded from the final analysis.

All data were collected using a standardized, anonymized electronic case report form. Postoperative complications were classified according to the Clavien–Dindo grading system, with major complications defined as Grade III or higher.

The primary end point was the identification of determinants of recovery on the third postoperative day. Postoperative recovery was defined as both the discontinuation of intravenous fluid therapy and the initiation of oral intake. These two items were chosen because the elements of postoperative recovery are inevitably interconnected. Achieving one goal facilitates the next: Removal of the nasogastric tube promotes the introduction of a liquid diet, which subsequently supports the transition to a solid diet and ultimately enables the discontinuation of intravenous fluid administration. Determinants of recovery on POD 3 were evaluated in the subgroup of patients without severe postoperative complications (Clavien–Dindo grade > IIIa), because the onset of major complications represents a strong determinant of delayed recovery and would have introduced a relevant confounding factor.

The secondary end point was the evaluation of the reasons for noncompliance with the postoperative protocol, which included a series of specific items assessed on predetermined postoperative days (PODs). On POD 1, pain control and the presence of a nasogastric tube (NGT) were evaluated: If pain was adequately managed and the NGT had been removed, patients were advanced to a liquid diet and encouraged to ambulate for more than 4 h. On POD 2, urinary output (> 0.5 mL/kg/h) and the absence of nausea or vomiting were assessed: If both conditions were met, a solid diet was initiated and the urinary catheter was removed. On POD 3, adequate oral intake, defined as tolerance of the prescribed diet, was evaluated: if achieved, intravenous fluids were discontinued. Postoperative compliance were recorded daily. In case of noncompliance to the proposed item. the reasons were also recorded: Postoperative complication, noncompliance to the requested condition, or protocol deviation.

### 2.1 Statistical Analysis

Continuous variables were summarized as medians with interquartile ranges (IQRs), whereas categorical variables were reported as absolute frequencies and percentages. Univariate analyses were conducted to identify associations between clinical variables and outcomes of interest, using the Mann–Whitney *U* test for continuous variables and the chi‐square or Fisher's exact test for categorical variables, as appropriate. Variables found to be statistically significant in univariate analysis (*p* < 0.05) were subsequently included in multivariate logistic regression models to identify independent predictors of delayed recovery. All statistical analyses were performed using IBM SPSS Statistics, Version 27 (IBM Corp., Armonk, NY, USA). A two‐tailed *p*‐value of < 0.05 was considered statistically significant.

## 3 Results

This is a secondary analysis of a previous multicenter study conducted between March 2023 and March 2024, which enrolled 760 patients. The characteristics of the study population and perioperative outcomes were summarized in Table 1.

**TABLE 1:** Patients’ characteristics and outcomes.

		*N*	%	Median	IQR
Age				70	56–80
Sex	F	390	51.3		
	M	370	48.7		
BMI				24	21–27
ASA	I and II	345	46.7		
	III and IV	394	53.3		
Charlson Comorbidty Index				4	2–6
Time from admission to surgery (hours)				8	3–24
Diagnosis	Intestinal obstruction	462	60.8		
	Intra‐abdominal infection	298	39.2		
Surgery	Enteric bypass	10	1.3		
	Lysis of adhesions	216	28.4		
	Resection with anastomosis	347	45.7		
	Resection without anastomosis	102	13.4		
	Hollow viscous perforation repair	85	11.2		
Surgical technique	Open	447	59.8		
	Successful laparoscopy	198	26.1		
	Conversion to open surgery	115	15.1		
Postoperative complications		266	35.0		
Clavien–Dindo grade	I	81	10.7		
	II	95	12.5		
	III	2	0.3		
	IIIa	17	2.2		
	IIIb	40	5.3		
	IVa	4	0.5		
	IVb	4	0.5		
	V	23	3.0		
SSI		74	10.0		
Anastomotic leak		22	5.2		
Respiratory complications		53	7.1		
Urinary tract infections		22	2.9		
Cardiovascular complications		39	5.2		
Reintervention		45	5.9		
Readmission		31	4.2		
Mortality		23	3.0		
Length of stay (days)				7	5–11

**FIGURE 1:**
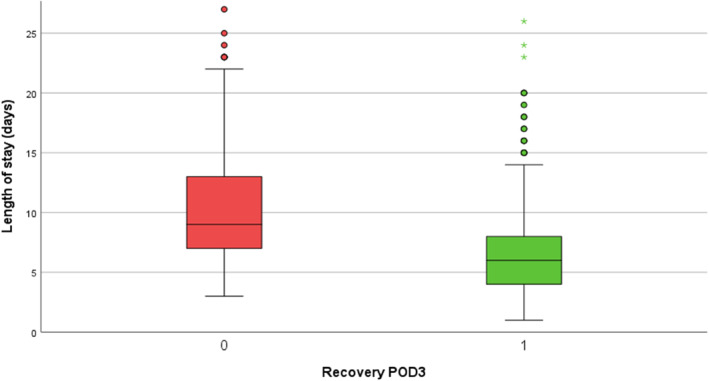
Postoperative length of stay.

Postoperative recovery, defined as the discontinuation of intravenous fluids and the initiation of oral intake, was achieved in 53.7% of patients by postoperative Day 3. Patients who did not achieve recovery by POD 3 had a significantly longer hospitalization, with a median length of stay of 9 days (IQR 7–13), than 6 days (IQR 4–8) in patients who reached the recovery targets (*p* < 0.001) as shown in Figure 1.

At multivariate analysis (Table 2), several factors were independently related with recovery on POD 3. Clinical frailty emerged as a significant negative predictor (OR 0.848, 95% CI 0.743–0.968, and *p* = 0.015). The absence of surgical drains (OR 1.855, 95% CI 1.252–2.748, and *p* = 0.003), intraoperative goal‐directed fluid therapy (GDFT) (OR 1.513, 95% CI 1.052–2.175, and *p* = 0.026), and anesthetic depth monitoring (OR 1.111, 95% CI 1.002–2.237, and *p* = 0.045) showed a positive relation with recovery.

**TABLE 2:** Determinants of recovery on POD 3.

	Univariate				Multivariate			
	OR	CI lower	CI upper	Sign.	OR	CI lower	CI upper	Sign.
Sex	1.263	0.931	1.713	0.134				
Age	0.976	0.967	0.986	< 0.001	0.987	0.973	1.002	0.095
BMI	1.003	0.972	1.036	0.837				
Clinical frailty	0.775	0.705	0.853	< 0.001	0.848	0.743	0.968	0.015
Charlson Comorbidity Index	0.917	0.869	0.969	0.002	1.036	0.952	1.126	0.416
ASA (III and IV)	2.323	1.696	3.181	< 0.001	0.643	0.422	0.980	0.040
Septic status	1.452	1.063	1.983	0.019	0.818	0.550	1.216	0.321
Lactate > 2	2.217	1.481	3.319	< 0.001	0.667	0.413	1.078	0.098
Timing of admission and intervention	1	0.997	1.003	0.994				
Lysis of adhesions				1				
Resection with anastomosis	1.047	0.877	2.038	0.065				
Resection without anastomosis	0.733	0.469	1.145	0.172				
Hollow viscous perforation repair	0.651	0.4	1.058	0.083				
Enteric bypass	0.431	0.11	1.694	0.228				
Anesthesia depth monitoring	1.645	1.181	2.291	0.003	1.503	1.010	2.237	0.045
Neuromuscular block monitoring	1.047	0.755	1.453	0.783				
PONV prevention	1.353	0.737	2.482	0.33				
Locoregional anesthesia	1.132	0.813	1.576	0.464				
Intraop. GDFT	2.024	1.045	2.053	< 0.001	1.513	1.052	2.175	0.026
Preop. GDFT	0.684	0.504	0.928	0.015	0.770	0.530	1.119	0.171
No morphine	1.907	1.168	3.115	0.01	1.447	0.845	2.479	0.178
Minimally invasive approach	2.033	1.424	2.904	< 0.001	1.422	0.910	2.223	0.122
No drain	2.098	1.514	2.909	< 0.001	1.855	1.236	2.783	0.003

An analysis of noncompliance with specific ERP protocol items revealed that protocol deviation for clinical decision‐making played a key role in 10%–15% patients, particularly regarding early oral intake and timely discontinuation of intravenous fluids (Table 3).

**TABLE 3:** Postoperative compliance to single items.

Item	Compliance	*N*	%
NG tube removal (POD 1)	Yes	489	65.9
	No, complication	19	2.5
	No, output > 300 mL	119	16.0
	Protocol deviation for medical decision	115	15.5
NG tube repositioning		84	11.6
Mobilization > 4h (POD 2)	Yes	540	73.5
	No, asthenia	92	12.5
	No, complication	24	3.3
	No, uncontrolled pain	10	1.4
	Protocol deviation for medical decision	69	9.4
Urinary catheter removal (POD 2)	Yes	431	58.5
	No, prolonged immobilization	94	12.8
	No, complication	30	4.1
	No, not adequate diuresis	68	9.2
	Protocol deviation for medical decision	113	15.3
Liquid diet (POD 2)	Yes	575	76.7
	No, complication	17	2.2
	Not tolerated	17	2.2
	Protocol deviation for medical decision	47	6.2
	No, NGT persistence	94	12.5
Stop IV fluids (POD 3)	Yes	438	58.9
	No, complication	47	6.3
	No, not adequate diuresis	28	3.8
	No, not adequate oral intake	134	18.0
	Protocol deviation for medical decision	97	13.0
Solid diet (POD 3)	Yes	494	65.9
	No, complication	38	5.0
	Liquid diet not tolerated	43	5.7
	Protocol deviation for medical decision	100	13.3
	No, NGT persistence	75	10.0

Noncompliance with NG tube removal on POD 1 was due to the persistence output greater than 300 mL (16%) and protocol deviation (15.5%). By the second postoperative day, 73.5% of patients were able to mobilize for more than 4 hours, with noncompliance primarily due to asthenia (12.5%) or medical decision (9.4%). Noncompliance with the urinary catheter on POD 2 was due to protocol deviation (15.3%) and scarce mobilization (12.8%). Also by the second day, 77% of patients resumed a liquid diet, with the persistence of the nasogastric tube being the main noncompliance reason (12.5%). By the third postoperative day, most patients (66%) have transitioned to a solid diet. Discontinuation of intravenous fluids was achieved in 59% of patients by the third day with inadequate oral intake as the major reason for noncompliance (18%).

## 4 Discussion

This secondary analysis of a multicenter prospective study shows that more than half of the patients treated within an enhanced perioperative care protocol after emergency laparotomy achieved recovery by (POD 3). These findings support and extend previous work suggesting that ERP principles can be successfully translated into emergency surgical care, achieving outcomes comparable to those observed in elective surgery populations [10, 11].

Several patient‐related and perioperative ERP items were found to significantly influence recovery pathways. Among them, clinical frailty emerged as a consistent and powerful predictor of delayed recovery, corroborating existing evidence on the importance of frailty assessment in surgical risk stratification [12]. Even in time‐sensitive contexts like emergency surgery, brief frailty screening tools may offer valuable prognostic insight and offer the possibility to manage patients with tailored perioperative strategies [13].

On the intraoperative side, several ERP process metrics were independently associated with faster recovery [14]. The depth of anesthesia monitoring and the use of goal‐directed fluid therapy both correlated with improved outcomes. These tools may contribute to the growing body of evidence that tailored fluid administration improves tissue perfusion and recovery trajectories [14], while also optimizing anesthetic depth, thereby reducing physiological stress and facilitating earlier functional recovery [15]. Additionally, the omission of surgical drains was consistently associated with faster recovery on POD 3. This finding aligns with accumulating literature suggesting that routine drain placement may be unnecessary in many abdominal procedures and may even delay mobilization, increase discomfort, and prolong hospitalization [16, 17].

Interestingly, although the minimally invasive surgical approach was not retained as an independent predictor in multivariate analysis, its positive impact in univariate models aligns with well‐documented benefits in terms of reduced postoperative pain, faster return of bowel function, and shorter hospital stays—even in emergency settings [18]. This supports existing literature that advocates for laparoscopic techniques in emergency settings when feasible [19]. Taken together, these results suggest that adherence to key enhanced perioperative care components can meaningfully influence recovery outcomes. Standardized implementation of such elements may help reducing the gap between elective and emergency perioperative care.

The recovery criteria used in this study—defined as discontinuation of intravenous fluids and full resumption of oral intake—are practical and clinically relevant milestones of enhanced perioperative care protocols. Their timely achievement in emergency settings has historically been questioned, yet our findings demonstrate higher rates of compliance than those reported in elective cohorts [20].

Patient‐level predictors of delayed recovery—including age, ASA score, and comorbidity burden—were consistent with known risk factors for adverse postoperative outcomes. However, their impact should not discourage the application of enhanced perioperative care pathways. These high‐risk profiles highlight the need for structured, evidence‐based perioperative care to reduce variability and optimize outcomes.

A key finding of this study is that protocol deviation due to clinical decision‐making accounted for a substantial proportion of noncompliance with postoperative ERP items. This observation deserves careful interpretation, particularly in the emergency setting. Although in elective surgery, enhanced recovery pathways are designed to guide perioperative care in a largely *proactive* manner—where protocol adherence is pursued systematically and deviations are exceptional—in emergency surgery, the application of ERP principles should instead be considered inherently *reactive*. In this context, the decision to implement each component of the pathway must follow a thorough and dynamic evaluation of the patient's physiological status, intraoperative findings, and early postoperative course. Predetermined clinical conditions—such as hemodynamic stability, adequate bowel function, absence of ongoing sepsis, acceptable aspiration risk, and overall functional reserve—must be satisfied before advancing through the recovery milestones. Consequently, deviations from the protocol frequently reflect appropriate and individualized clinical judgment rather than poor compliance. This distinction is crucial, as rigid application of ERP metrics in emergency settings risks oversimplify complex clinical scenarios and may inadvertently penalize surgeons for prudent decision‐making. The granularity of the collected (and collectable) data represents an important limitation. In the present study, reasons for noncompliance were recorded using predefined categories (postoperative complications, specific clinical conditions, or medical decision), and more detailed physiological or contextual determinants could not be systematically captured. As a result, the exact clinical rationale underlying each single deviation could not always be fully elucidated. This limitation should be acknowledged when interpreting protocol adherence metrics and further highlights the complexity of applying standardized recovery pathways in heterogeneous emergency surgical populations. Enhanced recovery pathways in emergency surgery should therefore be interpreted as flexible, evidence‐based frameworks that support, rather than replace, surgeon expertise, with the ultimate goal of optimizing patient safety and recovery while preserving individualized care.

Moreover hospital staff in the emergency setting could vary enormously, with lack of a dedicated and trained staff. Although individualized care remains essential, our findings suggest that improved adherence to selected ERP practices, even in the face of uncertainty, may contribute to better early recovery. Educational interventions, multidisciplinary buy‐in, and continuous audit–feedback loops may support culture change in this direction [21].

This study has some limitations, which should be acknowledged. Recovery was defined using two binary end points—resumption of oral intake and cessation of intravenous fluids—which may not fully capture functional recovery or patient‐reported outcomes. Additionally, although major complications were excluded from the primary analysis, residual confounding from minor or subclinical adverse events cannot be entirely excluded. Nonetheless, the study has several strengths, including its prospective and multicenter design, robust sample size, and adjustment for multiple confounders.

## 5 Conclusion

The results of this study suggest that early recovery after surgery is influenced both by intrinsic patient factors and by adherence to ERP strategies. Although challenges remain in ensuring compliance to the postoperative recovery pathway, this study underscores the potential of ERP to improve perioperative care in the emergency setting. However, the study also highlights the need of targeted education, continuous audit, and systematic quality improvement initiatives to improve the ERP adherence. Future research should further clarify how these determinants interact and whether targeted interventions could mitigate the higher risk of delayed recovery.

## Author Contributions

**Marco Ceresoli:** conceptualization, investigation, writing – original draft, methodology, writing – review and editing, data curation, formal analysis. **Chiara Fumagalli:** investigation, writing – original draft, methodology, data curation, formal analysis. **Alan Biloslavo:** investigation, writing – review and editing. **Antonio La Greca:** investigation, writing – review and editing. **Michele Carlucci:** investigation, writing – review and editing. **Giovanni Pesenti:** investigation, writing – review and editing. **Savino Occhionorelli:** investigation, writing – review and editing. **Pietro Bisagni:** investigation, writing – review and editing. **Carlo Feo:** investigation, writing – review and editing. **Dario Tartaglia:** investigation, writing – review and editing. **Dario Parini:** investigation, writing – review and editing. **Matteo Runfola:** investigation, writing – review and editing. **Riccardo Somigli:** investigation, writing – review and editing. **Diego Visconti:** investigation, writing – review and editing. **Diego Mariani:** investigation, writing – review and editing. **Diletta Cassini:** investigation, writing – review and editing. **Andrea Mingoli:** validation, visualization, supervision, writing – review and editing. **Marco Braga:** conceptualization, investigation, supervision, writing – review and editing.

## Funding

The authors have nothing to report.

## Ethics Statement

The study protocol was approved by the local ethic review board.

## Consent

Informed consent was obtained from all individual participants included in the study.

## Conflicts of Interest

The authors declare no conflicts of interest.

## Supporting information


Supporting Information S1

## Data Availability

The data that support the findings of this study are available from the corresponding author upon reasonable request.
