# Supplementary material for: Determinants of Compliance to Enhanced Recovery Protocol After Emergency Laparotomy
Source: World J Surg. 2026 Mar 25;50(5):1213–20. doi: 10.1002/wjs.70312 (PMC13206338; doi:10.1002/wjs.70312)
Supplement: Supplementary file 1 — Supporting Information S1 [file WJS-50-1213-s001.docx]

**Collaborative group:**

Marco Cereda^1,2^, Luca Gianotti^1,2^, Luca Nespoli^1,2^, Michele Rossi^2^, Mauro Zago^6^, Michele Fogliata^2,6^, Elisa Bolzoni^6^, Giuseppe Foti^1,18^, Michele Ballabio^8^, Vera D'Abrosca^8^, Gianluca Russo^23^, Carmine Votta^23^, Antonella D’addiego^5^, Enrico Ortolano^5^, Aldo Alberto Beneduce^5^, Luca Ghirardelli^5^, Valeria Fico^4^, Valerio Cozza^4^, Paolo Mirco^4^, Silvia Tedesco^4^, Fausto Rosa^4^, Nicolò Fabbri^9^, Antonio Pesce^9^,Luca Bagnoli^24^, Jessica Polito^24^, Mauro Santarelli^14^, Roberta Tutino^14^, Elena Herranz van Nood^14^, Angelica Vadagnini^14^, Domenico Lacavalla^7^, Rocco Stano^7^, Dario Andreotti^7^, Maria Grazia Sibilla^7^, Irene Lorenzi^25^, Federico Coccolini^10^, Massimo Chiarugi^10^, Francesco Giudice^10^, Silvia Strambi^10^, Elisa Boetti^13^, Paola Germani^3^, Sara Crociato^3^, Enrico Lena^19^, Romina Manunza^12^, Federica Frongia^12^, Marcello Agus^12^, Elisabetta Pusceddu^22^, Francesco Murgia^22^, Andrea Russo^26^, Giorgia Boschetto^20,21^, Daria Zanirato^11^, Renza Zeggio^11^, Gianandrea Baldazzi^15^, Marta Spalluto^15^, Paolo Pizzini^15^, Giovanni Tarchi^15^, Lorenzo Guiotto ^27^, Claudia Zaghi^28^

Affiliations

1: University of Milano-Bicocca, School of Medicine and Surgery, Monza, Italy

2: General and Emergency Surgery, Fondazione IRCCS San Gerardo dei Tintori, Monza, Italy

3: Department of General Surgery, Cattinara University Hospital, ASUGI, Trieste

4: Dip.to Scienze Mediche e Chirurgiche, UOC Chirurgia d'Urgenza e Trauma, Fondazione Policlinico Universitario A. Gemelli IRCCS Roma - Università Cattolica del Sacro Cuore

5: Department of Emergency and General Surgery, IRCCS San Raffaele Hospital, Milan

6: UOC Chirurgia Generale e d'Urgenza, Dip. chirurgico, H. A. Manzoni,  ASST Lecco

7: s.s.d. Emergency surgery, Surgical Department, University of Ferrara, Arcispedale S. Anna di Cona

8: Dept of Surgery, ASST Lodi, Lodi, Italy

9: UOC Chirurgia Generale Provinciale, Azienda USL di Ferrara - Università di Ferrara

10: General, Emergency Surgery Unit and Trauma Center, Pisa University Hospital, Pisa

11: General Surgery Department, Santa Maria della Misericordia Hospital, Rovigo - ULSS 5

12: General and Emergency Surgery Unit, Dept of Emergency, ARNAS “G. Brotzu” , Cagliari, Italy

13: dipartimento Chirurgia usl toscana centro- ospedale Santa Maria Nuova

14: Chirurgia Generale d’Urgenza e PS, AOU Città della Salute e della Scienza, Torino

15: General Surgery, Ospedale di Legnano, ASST ovest Milanese, Legnano, Italy

16: General Surgery, ASST Milano-Nord, Sesto San Giovanni, Italy

17: president of the Italian Society of Emergency Surgery and Trauma (SICUT)

18: department of Anesthesia and Intensive Care, Fondazione IRCCS San Gerardo dei Tintori, Monza

19: Department of Anesthesiology and Critical Care, Cattinara University Hospital, ASUGI, Trieste

20: Anesthesia and Intensive Care Unit, Santa Maria della Misericordia Hospital, Rovigo -ULSS 5

21: Anesthesia and Intensive Care Unit, Maurizio Bufalini Hospital, Cesena - Ausl Romagna

22: Anesthesia and Intensive Care Unit, Liver Transplantation Center, ARNAS “G. Brotzu”, Cagliari, Italy

23: Anesthesiology and Critical Care dept., ASST Lodi, Lodi Italy

24: UOC Anestesia e Rianimazione, Ospedale del Delta, Azienda USL di Ferrara

25: Anesthesiology and Critical Care Unit, Pisa University Hospital, Pisa

26: Dip.to Scienze dell'Emergenza, Anestesiologiche e della Rianimazione, UOC Anestesia delle Chirurgie Generali e dei Trapianti, Fondazione Policlinico Universitario A. Gemelli IRCCS Roma

27: Anesthesia and Intensive care Unit - H A. Manzoni, ASST Lecco, Lecco Italy

28: General Surgery, Ospedale San Bortolo, ULSS Berica, Vicenza, Italy
